# Implementation of enhanced recovery after surgery (ERAS) protocols in elderly patients undergoing emergency surgery for perforated peptic ulcer: a comparative analysis

**DOI:** 10.1186/s12876-025-04188-0

**Published:** 2025-10-13

**Authors:** Mohamed Wael, Ahmed S. Shehab, Islam El-Sayes, Mostafa Ibrahim Ahmed Seif-Eldeen

**Affiliations:** 1 Alexandria Main University Hospital, Alexandria, Egypt; 2 Department of Surgery, Faculty of Medicine, Alexandria University, Alexandria, Egypt; 3 Anaesthesia and surgical intensive care department, Faculty of Medicine, Alexandria University, Alexandria, Egypt

**Keywords:** ERAS, Elderly, Perforated peptic ulcer, Perioperative care, Emergency surgery

## Abstract

**Background:**

Enhanced Recovery After Surgery (ERAS) protocols are well-established in elective surgery, yet their role in emergency procedures remains underexplored — particularly in the elderly, a uniquely vulnerable subgroup. This study presents the first focused comparative analysis evaluating ERAS outcomes in elderly patients undergoing emergency surgery for perforated peptic ulcer (PPU).

**Methods:**

This retrospective comparative study analyzed 137 elderly patients (≥ 60 years) who underwent emergency surgery for PPU from August 2020 to July 2024. Patients were divided into: ERAS group (*n* = 67) and conventional group (*n* = 70), based on their perioperative protocol of management. Primary outcomes included postoperative functional recovery parameters. Secondary outcomes encompassed postoperative complications, pain scores, length of hospital stay and readmission rates.

**Results:**

The ERAS group demonstrated significantly faster functional recovery across multiple parameters: earlier bowel movement (1.21 ± 0.34 vs. 2.20 ± 0.57 days, *p* = 0.008), faster mobilization (1.26 ± 0.17vs 3.51 ± 0.60 days, *p* < 0.001), and shorter hospital stay (5.24 ± 0.57 vs. 7.03 ± 2.09 days, *p* = 0.001). Pain control was superior in the ERAS group, with consistently lower VAS scores and reduced opioid consumption (8.49 ± 0.59 vs. 18.73 ± 0.72 mg, *p* = 0.001). Notably, postoperative nausea and vomiting were significantly reduced (22.4% vs. 41.8%, *p* = 0.013). Complication rates were comparable between groups, with no increase in readmission and reoperation rates, suggesting the safety of ERAS implementation.

**Conclusion:**

This focused analysis provides evidence that ERAS protocols can be safely and effectively implemented in elderly patients undergoing emergency PPU repair, resulting in accelerated recovery, improved pain control, and reduced hospital stay without compromising safety. These findings challenge traditional conservative approaches to perioperative care in elderly emergency surgery patients. However, our findings primarily apply to a relatively stable ‘young-elderly’ subgroup and may not reflect outcomes in frailer or critically ill elderly populations.

## Background

Perforated peptic ulcer (PPU) represents a severe complication of peptic ulcer disease, resulting in the spillage of gastric or duodenal contents into the peritoneal cavity necessitating a surgical repair [1, 2].

Traditionally, the perioperative management of PPU relied on extended fasting, delayed mobilization and heavy opioid -based analgesia. This led to unfavorable outcomes including infections, delayed restoration of peristalsis and extended hospital stay [3].

Accordingly, Enhanced Recovery After Surgery (ERAS) protocol has evolved as a comprehensive, evidence-based strategy for perioperative care. Its main goal is to minimize the physiological and psychological stress linked to surgical interventions, thereby shortening the time to complete recovery. Essential elements of ERAS encompass reduced preoperative fasting, early introduction of oral nutrition, improved pain control strategies, and facilitating prompt postoperative mobilization [4].

Elderly individuals with PPU disease face unique challenges; patients tend to have a late presentation which is further exacerbated by diminished immune response, reduced cardiovascular and respiratory capacity, and compromised wound healing. Collectively, these elements lead to increased incidences of postoperative complications and mortality rates in this age group [5].

While ERAS has shown benefits in elective and some emergency surgical procedures, limited data exist on its application specifically in the elderly population undergoing emergency surgery for PPU. Most existing studies either focus on mixed-age populations or do not report elderly-specific outcomes. Moreover, few studies provide comprehensive recovery metrics such as early ambulation, opioid-sparing analgesia, and gastrointestinal function recovery in this context [6–8].

Although multiple studies were published regarding the feasibility of applying ERAS protocol in the elderly population and in PPU surgery, this is a novel comparative study to provide a focused evidence on applying ERAS protocol specifically in elderly patients undergoing emergency surgery for PPU, evaluating both functional recovery and safety. The primary objective of this study is to compare the outcomes of Enhanced Recovery After Surgery (ERAS) versus conventional perioperative care in elderly patients undergoing emergency laparoscopic repair of PPU. The study aimed to evaluate functional recovery, complication rates, pain control, and hospital stay between the two groups.

## Materials and methods

### Patient selection

From August 2020 to July 2024, collected data from our records for patients older than 60 years who presented to our facility and underwent laparoscopic repair of PPU were retrospectively analyzed. This age threshold was chosen to include early geriatric patients and to improve the sample size, consistent with definitions used in global health and some surgical aging studies. The study was approved by the ethics committee and review board of Alexandria Main University Hospital, Egypt.

Patients were allocated to either the ERAS or conventional group based on a time-based, non-randomized protocol implementation model. Between August 2020 and July 2022, conventional perioperative care was the traditional method of managing PPU repair patients in our institution. Starting from August 2022, ERAS protocol was introduced at our center for PPU repair including elderly patients. Thus, patients who underwent surgery before the ERAS implementation were assigned to the conventional group, while those treated after protocol application were managed under the ERAS pathway. This time-sequential allocation reduced potential selection bias and allowed for consistent protocol application within each group.

Based on this allocation method, Patients were subdivided into two groups based on the application of ERAS protocol in their perioperative management: (a) **The ERAS group**: elderly patients managed with perioperative ERAS protocol, (b) **The Conventional group**: elderly patients managed with standard perioperative care.

This study focused on hemodynamically stable elderly patients with PPU who were candidates for laparoscopic repair. The following patients were excluded; Age < 60 years, chronic steroid use, malignancy confirmed via ulcer margin biopsy, patients with deviation from the ERAS protocol due to different causes, patients converted to open repair and patients with refractory septic shock (serum lactate > 4 mmol/L despite resuscitation).

### Perioperative Preparation and intraoperative care

Patients in both groups received standard preoperative care, including Nasogastric (NG) suction, urethral catheterization, intravenous crystalloids, parenteral broad-spectrum antibiotics and blood transfusion requested for correction of anemia according to Hemoglobin level. Baseline investigations included ECG, echocardiography, routine laboratory assessment and arterial blood gas. Diagnosis was confirmed with abdominal CT scan.

Propofol was mainly used for induction with age-adjusted dose (1-1.5 mg/kg). Maintenance was carried out in the ERAS group with sevoflurane with MAC values of 0.5–0.7(, While in the conventional group, isoflurane with higher MAC values (1.0-1.2) was used.

In the ERAS group, multimodal analgesia (intravenous paracetamol, NSAIDs unless contraindicated, low-dose ketamine (0.1–0.2 mg/kg), TAP (Transversus Abdominis Plane) block with rectus sheath block and wound infiltration) was implemented. While in the conventional group, a less structured multimodal primarily opioid-based intraoperative analgesia approach was adopted with higher doses of Fentanyl (1–2 mcg/kg).

Epidural analgesia was used as needed. Muscle relaxation was achieved using rocuronium, reversed by sugammadex in the ERAS group. While in the conventional group, any type of muscle relaxant (reversed with atropine and neostigmine) was used. Hypothermia and swinging of hemodynamics were prevented with good glycemic control in both groups.

### Surgical technique

Exploratory laparoscopy and repair of the PPU site with omental patch was carried out. After thorough peritoneal lavage, abdominal drains were placed in the subhepatic and pelvic spaces. Patients with intraoperative findings of spontaneously sealed off perforated ulcers were excluded from analysis.

### Postoperative care

According to hemodynamics, laboratory tests, level of consciousness and respiratory mechanics; patients of both groups were transported after full reversal of neuromuscular blockade to either post anesthesia care unit (PACU) or surgical ward.

Regarding postoperative pain management, pain was assessed using Visual Analog Scale (VAS) from 0 to 10 at 24 h intervals postoperatively. In the ERAS group, non-opioid multi-modal analgesia was used. Regular medications involved scheduled IV/oral Paracetamol with dose adjusted according to the age and body weight (650 mg q6h) and NSAIDs (unless contraindicated). Opioid was used only for breakthrough pain attacks if the VAS score ≥ 4. If needed, low dose short acting opioids were used (Fentanyl 25 µg). In the conventional group, regular opioids were used with age adjustment (low dose, long intervals of Meperidine 25 mg) if the VAS score ≥ 4 with less emphasis on regional analgesia and more reliance on systemic medications. All patients were treated with Ondansetron 4 mg twice daily to guard against postoperative nausea and vomiting.

In the ERAS group, early same-day postoperative ambulation was encouraged. If an epidural catheter was inserted, sitting for 2–3 h on the day of surgery and ambulation after removal was practiced. In the conventional group, patients were encouraged for early ambulation according to their pain tolerance and time of removal of tubes and drains.

In the ERAS Group, urinary catheter was removed once adequate urinary output and cardiovascular stability were achieved. NG tube was removed once the amount of daily effluent was accepted and/or peristalsis was audible. Abdominal drains were removed once the effluent decreased in daily amount and proved to be non-suspicious. In the conventional Group, urinary catheter was removed on the POD 2–3. NG tube was removed after passage of flatus. Abdominal drains were removed after the patient safely started soft oral intake.

Oral intake was started once peristalsis was heard in the ERAS group. Whereas, it was started after passage of flatus and removal of NG tube in the conventional group.

All patients were discharged when clinically stable after successfully tolerating a solid diet for a minimum of 24 h, having passed stool. Each patient received oral omeprazole 40 mg twice daily for at least 3 months. Patients in both groups were followed for 1 month in the outpatient clinic.

### Outcome measurements

The primary outcome was to compare the effect of applying the ERAS protocol on postoperative functional recovery metrics in elderly patients undergoing laparoscopic repair for PPU. Secondary outcomes included the effect of applying the ERAS protocol on the postoperative hospital stay, complication rate, maximum pain score and readmission rate.

All patients in the both groups included in the final analysis completed the protocol as designed. No major deviations were observed in any of the key components, including early feeding, ambulation, opioid-sparing analgesia, and early removal of tubes and drains.

Functional recovery was assessed based on early postoperative milestones, including time to first ambulation, flatus, and bowel movement. Validated scales for functional independence, such as the Barthel Index or ADL scoring, were not applied due to the retrospective nature of the study.

### Sample size

Sample size was calculated based on a previous study and by using Med Calc statistical software. Assuming an alpha of 0.05 and power of study 90.0%. A typical advice is to reject the null hypothesis H0 if the corresponding p-value smaller than 0.05. a minimum sample size required was 120 patients will be required for this study, 60 patients in at least each group.

### Statistical analysis

IBM SPSS Statistics for Windows, version 25.0 was used to perform different statistical analysis. Means and standard deviations were used to summarize numerical variables and counts and percentages were used to summarize categorical variables. The student’s t-test was used to assess numerical data that followed a normal distribution. The Mann-Whitney test was used for numerical variables that did not follow a normal distribution. Categorical variables were compared with the chi-square test or Fisher’s exact test. P-value of ≤ 0.05 were regarded as significant.

## Results

**Fig. 1:**
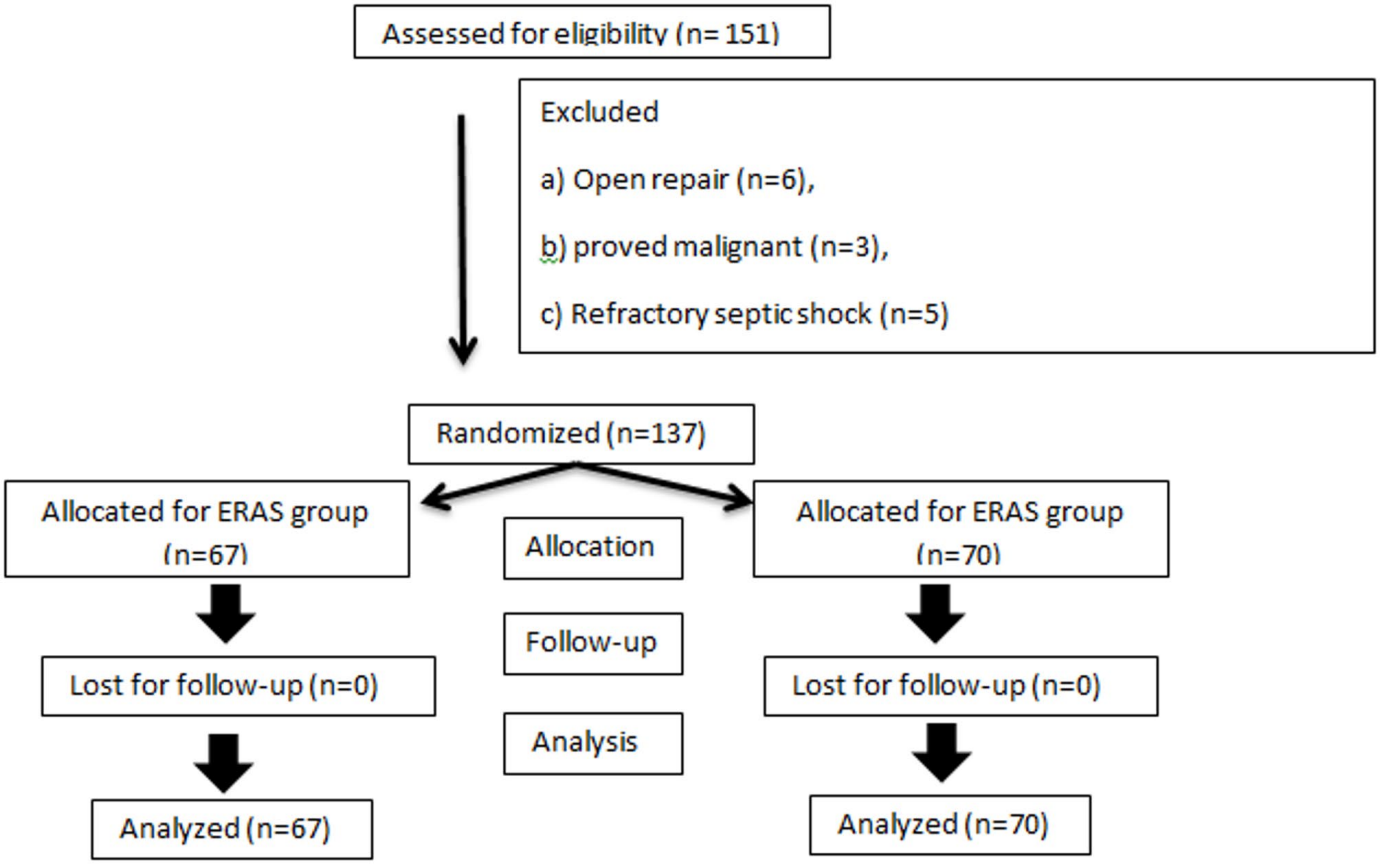
CONSORT-style flowchart diagram of the study

During the study period, a total of 151 elderly patients underwent emergency surgery for PPU and were assessed for eligibility. Fourteen patients were excluded from further analysis (6 patients needed open intervention, 3 patients proved to have underlying malignancy and 5 patients due to refractory septic shock). A CONSORT-style flowchart diagram is shown in Fig. 1.

Demographics, American Society of Anesthesiologists (ASA) score and associated comorbidities: There were no significant differences between the ERAS (*n* = 67) and conventional (*n* = 70) groups as shown in Tables 1 and 2.

**Table 1:** Baseline characteristics of ERAS vs. conventional-care patients

Variable	The ERAS group “*n* = 67”			The Conventional group “*n* = 70”			*p*-value
	No.		%	No.		%	
**Age (years)**							
Range Mean ± SD	60–79 68.70 ± 5.97			62–78 68.5 ± 4.6			0.406
**Gender**							0.606 0.435
Male	30		44.8	36		51.4	
Female	37		55.2	34		48.6	
**BMI (Kg/m** ^**2**^ **)**							
Range Mean ± SD	22.5–29 26 ± 1.9			23–30 26.5 ± 2.5			0.103
**ASA score**							
Class 1	31	46.3		29	41.4		0.535 0.765
Class 2	32	47.8		35	50		
Class 3	4	6		6	8.6		

Continuous variables are presented as mean ± standard deviation

Categorical variables are presented as number (percentage)

*P* < 0.05 was considered statistically significant

ASA: American Society of Anesthesiologists score, BMI: body mass index

**Table 2:** Comparison between the two studied groups regarding comorbidity

	The ERAS group	The Conventional group	*p*-value
	“*n* = 67”	“*n* = 70”	
	No. (%)	No. (%)	
Diabetes	17(25.4%)	19(27.1%)	0.452
Hypertension	9(13.4%)	13(18.6%)	0.338
Thyroid disorder	4(6%)	2(2.9%)	0.276
Chronic kidney disease	3(4.5%)	2(2.9%)	0.166
Cirrhosis	3(4.5%)	5(7.1%)	0.762
Ischemic heart disease	2(3%)	4(5.7%)	0.815
Obstructive pulmonary disease	4(6%)	2(2.9%)	0.276
Osteoporosis	2(3%)	0%	0.188
Bleeding disorder	1(1.5%)	2(2.9%)	0.347

*P* < 0.05 was considered statistically significant

Categorical variables are presented as number (percentage)

Duration of illness and intraoperative characteristics were comparable between both groups (*P* ≥ 0.05) (Table 3).

**Table 3:** Comparison between the two studied groups regarding duration of illness and intraoperative characteristics

Variable	The ERAS group “*n* = 67”		The Conventional group “*n* = 70”		*p*-value
	No	(%)	No	(%)	
**Duration of illness (hours)** Range Mean ± SD	35–55 45 ± 5.9		34–54 44.4 ± 6.7		0.277
**Size of perforation** ** <**5 mm 5–10 mm >10 mm	12 32 23	17.9 47.8 34.3	14 30 26	20.0 42.9 37.1	0.336 0.845 0.544
**Type of peritoneal collection**					
Biliary	20	30	22	31	0.040
Purulent	47	70	48	69	0.841

Continuous variables are presented as median ± standard deviation

Categorical variables are presented as number (percentage)

*P* < 0.05 was considered statistically significant

**Anesthetic agents, fluid administration and postoperative analgesia:** The ERAS group demonstrated significant improvements in anaesthesia and intraoperative analgesia management compared to the conventional group.

a) Intraoperative analgesia: A multimodal analgesia approach in the ERAS group showed a reduced opioid use (Fentanyl: 84.5±5.5μg vs. 155±6μg) (*p* =0.001).

b) Muscle relaxants and reversal: a significantly shorter time to emergence was noted in the ERAS vs. conventional group (15.2±0.5 min vs. 22.8±0.5 min, *p*= 0.003).

c) Fluid Management: Intraoperative fluid administration was significantly reduced in the ERAS group (1657.5±59 mL vs. 2448.7±62.2 mL, *p*= 0.004) to align with goal-directed fluid therapy principles.

**Functional postoperative recovery parameters:** The ERAS group demonstrated significantly faster functional recovery across multiple parameters, as shown in Table 4.

While several recovery milestones were achieved earlier in the ERAS group, some outcomes such as NG tube and catheter removal were predefined elements of the ERAS protocol and represent process measures rather than spontaneous physiologic milestones.

**Table 4:** Comparison between the two studied groups regarding postoperative functional recovery parameters

Time of functional postoperative parameter (days)	The ERAS group “*n* = 67”	The Conventional group “*n* = 70”	*p*-value
Time to audible bowel sounds	1.2 ± 0.3	2.2 ± 0.6	0.001
Time to pass flatus	2.3 ± 0.6	2.5 ± 0.6	0.027
Time to pass stools	2.9 ± 0.6	4 ± 0.6	0.002
Time to start oral fluids	1.5 ± 0.4	3.3 ± 0.5	0.003
Time to first semisolid food	3.2 ± 0.6	4.4 ± 0.5	0.004
Time to first ambulation	1.3 ± 0.2	3.5 ± 0.6	0.005
Time of nasogastric tube removal	1.4 ± 0.3	3.5 ± 0.5	0.002
Time of urinary catheter removal	1.6 ± 0.4	2 ± 0.6	0.001
Time of drain removal	2.9 ± 0.6	5 ± 1.4	0.003
Length of hospital stay	5.2 ± 0.6	7 ± 2	0.001

Values presented in mean ± SD

Statistically significant when the P-value is less than 0.05

### Postoperative outcomes

a) Postoperative nausea and vomiting showed significantly lower overall incidence in the ERAS group (22.4% vs. 41.8%, *p*=0.013). The need for rescue antiemetic medications was significantly lower in the ERAS group (17.9% vs. 37.3%, *p*=0.021).

b) Complications: Superficial wound infection occurred in 8 patients (12%) in the ERAS group vs. 11 patients (16%) in the conventional group (*p*=0.211). All patients were treated with regular dressing. Unfortunately, 1 patient in the ERAS group experienced leakage, while none occurred in the conventional group. This patient was a 69-year-old female with a 15 mm perforated duodenal ulcer who developed a fever on the third postoperative day. An abdominal ultrasound indicated a mild collection, and leakage was confirmed through an oral contrast CT scan. A re-laparotomy was performed. Additionally, postoperative ileus was noted in 1 patient from the conventional group, who was managed with bowel rest, reinsertion of a nasogastric tube, and correction of hypokalemia, with bowel feeding resumed upon improvement. Myocardial infarction occurred in 1 patient in the ERAS group who was managed by adequate oxygenation, aspirin and nitroglycerin. There was no significant difference between both groups regarding the postoperative complications. Interestingly, no pulmonary complications occurred in either group in the early postoperative period.

c) Reoperation: 1 patient in each group was re-operated (*p*= 0.98). One patient was operated in the ERAS group for leakage with re-closure of the perforated ulcer. One patient in the conventional group was re-operated three weeks postoperative for adhesive intestinal obstruction.

d) Readmission: A total of 3 patients required hospital readmission within 30 days of discharge. In the conventional group, one patient was readmitted on postoperative day 6 with an acute deep vein thrombosis, likely precipitated by reduced mobility and delayed ambulation during the early postoperative period. Another patient from the same group was readmitted on day 13 due to hospital-acquired pneumonia that developed after prolonged bed rest and delayed respiratory physiotherapy. In the ERAS group, one patient was readmitted on day 21 with symptoms of adhesive intestinal obstruction. All cases were considered related to the index surgery or its sequelae. The 30-day readmission rate was 2.9% in the ERAS group versus 5.3% in the conventional group (*p* = 0.621).”

## Discussion

This study addresses a unique and underexplored area; the implementation of ERAS protocols in elderly patients undergoing emergency repair of PPU. While previous literature has examined ERAS in elective or mixed-emergency cohorts [9–11], few have focused exclusively on elderly patients—a group known to have higher surgical risk, delayed recovery, and increased opioid sensitivity. By focusing on detailed recovery parameters such as early mobilization, bowel function, and pain scores, our study offers a novel and focused contribution that builds on prior ERAS research and informs practice for elderly surgical care. Our study demonstrates that ERAS protocols can be safely and effectively implemented in this high-risk population, with significant improvements in various outcome measures.

The demographic characteristics and comorbidity profiles were comparable between our study groups. This homogeneity strengthens the validity of our findings and aligns with recent studies by Chndan, Khakholia [3] who reported similar baseline characteristics in their randomized study comparing ERAS versus standard care in emergency surgery for PPU. Although we used 60 years as the cutoff for defining ‘elderly,’ the average age of participants was approximately 68 years. This suggests that the majority of patients in our cohort fall into the ‘young-elderly’ category, with few octogenarians included. Therefore, caution should be taken when generalizing these findings to very elderly or frail patients.

The tailored anesthesia and analgesia strategies employed in the ERAS group significantly contributed to the improved recovery outcomes observed in this study. Short-acting anesthetic agents, such as Sevoflurane, were used at lower MAC values in the ERAS group. This minimized residual anesthetic effects and enabled faster emergence from anesthesia and earlier mobilization, compared to the conventional group, which predominantly used Isoflurane.

Additionally, the incorporation of multimodal analgesia—including NSAIDs, paracetamol, and low-dose ketamine—resulted in significantly lower opioid consumption in the ERAS group (8.49 ± 0.59 mg vs. 18.73 ± 0.72 mg, *p* = 0.001), effectively minimizing opioid-related side effects such as nausea and vomiting (22.4% vs. 41.8%, *p* = 0.013) and delayed gastrointestinal recovery.

The frequent use of regional techniques, such as transversus abdominis plane (TAP) blocks and rectus sheath blocks, provided effective pain control, allowing for earlier mobilization and improved patient comfort. In contrast, the conventional group relied heavily on opioids, resulting in higher pain scores (VAS at 24 h: 2.00 ± 0.78 vs. 3.44 ± 1.21, *p* = 0.001) and a greater incidence of postoperative nausea and vomiting. These findings underscore the critical role of optimized anesthesia and analgesia in enhancing postoperative recovery and highlight the importance of integrating these strategies into ERAS protocols, particularly for high-risk emergency surgical procedures.

The significantly reduced fluid administration in the ERAS group (1657.55 ± 58.97mL vs. 2448.7 ± 62.18mL, *p* < 0.001) reflects modern goal-directed fluid therapy principles. This approach is supported by Aggarwal, Peden [12], who demonstrated that restricted fluid protocols reduce complications and enhance recovery in emergency abdominal surgery.

Early mobilization is an important component of ERAS protocols. Prolonged bed rest increases pulmonary complications, thromboembolism, insulin resistance and decreases muscle strength [13]. Early mobilization, achieved significantly earlier in the ERAS group (1.26 ± 0.17 days vs. 3.51 ± 0.60 days, *p* = 0.005), represents a crucial component of the protocol. This finding aligns with research by Juita, Yona [14], showing that early mobilization reduces pulmonary complications and accelerates functional recovery after abdominal surgery. Furthermore, Schaller, Anstey [15] conducted a multicenter study showing benefit of early mobilization in surgical ICU patients; length of stay was shortened and patients’ functional ability at discharge was increased.

The earlier removal of nasogastric tubes (1.37 ± 0.34 vs. 3.45 ± 0.54days, *p* = 0.002) and urinary catheters (1.64 ± 0.39vs 2.06 ± 0.64days, *p* = 0.001) in the ERAS group represents a significant advancement in postoperative care. This approach is supported by the meta-analysis published by Zeyara, Thomasson [16], showing that early removal of drains and tubes does not increase complications and may reduce the risk of hospital-acquired infections. This correlates with the ERAS society guidelines who recommended early removal of nasogastric tubes and urinary catheters in patients undergoing emergency laparotomy [17]. 

The early initiation of oral intake in the ERAS group was a pivotal component of improved postoperative recovery which was evident across all measured parameters in this study. Patients in the ERAS group were able to resume oral fluid intake significantly earlier (1.52 ± 0.36 days) compared to the conventional group (3.25 ± 0.54days), *p* = 0.003. Early feeding not only reduced the duration of fasting but also contributed to faster recovery of gastrointestinal function, as evidenced by the earlier return of bowel sounds (1.21 ± 0.34 days vs. 2.20 ± 0.57days, *p* = 0.001), time to first flatus (2.34 ± 0.57 vs. 2.53 ± 0.59 days, *p* = 0.027) and quicker time to first stools (2.92 ± 0.62 days vs. 3.96 ± 0.60 days, *p* = 0.002). This aligns with the randomized study by Klappenbach, Yazyi [18] that showed no increase in complications with feeding within 24 h of emergency surgery compared with a traditional approach of starting with a liquid diet after passage of flatus or stool. In contrast, delayed oral intake in the conventional group prolonged gastrointestinal recovery and was associated with a higher incidence of postoperative nausea and vomiting (22.4% vs. 41.8%, *p* = 0.013). The findings are consistent with those reported by Gonenc, Dural [19], who demonstrated similar improvements in gastrointestinal function recovery with ERAS protocols in emergency abdominal surgery and reinforce the safety and efficacy of early feeding as part of ERAS protocols, challenging the traditional practice of prolonged fasting in emergency surgical settings. By mitigating catabolic stress and improving patient comfort, early feeding serves as a cornerstone for faster recovery and reduced length of hospital stay.

It is important to differentiate between protocol-driven process outcomes and true functional recovery. For example, early NG tube and catheter removal, and early feeding initiation, were part of the ERAS protocol by design. Their successful implementation without increased rates of ileus or infection supports the safety of these accelerated interventions, rather than their spontaneous occurrence. Furthermore, claims of ‘faster return of bowel function’ should be interpreted as partially protocol-enabled rather than entirely patient-driven, highlighting the importance of cautious interpretation.

Pain management showed marked improvement with the ERAS protocol, with consistently lower VAS scores and significantly reduced opioid consumption. This multimodal approach to analgesia, emphasizing regional techniques and non-opioid alternatives, mirrors the findings of El-Kefraoui, Do [20], who reported superior pain control and reduced opioid requirements with similar protocols in emergency laparotomy.

PONV is a major cause of patient dissatisfaction and delays return to enteral intake. All patients undergoing emergency laparotomy are at high risk of PONV due to physiological derangement and gastrointestinal insult. The lower incidence of PONV in the ERAS group is consistent with the findings of the ERAS^®^ Society guidelines published by Feldheiser, Aziz [21], who emphasized the importance of multimodal PONV prophylaxis in ERAS protocols. This improvement likely results from combination of reduced opioid use, early oral intake, and targeted antiemetic prophylaxis.

The reduction in length of hospital stay (5.24 ± 0.57 vs. 7.03 ± 2.09days, *p* = 0.001) is particularly noteworthy and aligns with findings published by Paduraru, Ponchietti [22], who conducted a systematic review showing significant reductions in hospital stay with ERAS protocols in emergency surgery. This decrease in hospitalization duration not only reduces healthcare costs but may also lower the risk of hospital-acquired and reduced mobility complications in this vulnerable elderly population.

Importantly, our study showed no increase in complications or readmission rates with the ERAS protocol, despite earlier discharge and faster recovery trajectories. This safety profile is consistent with systematic reviews by Tengberg, Bay-Nielsen [23], who found that ERAS protocols do not increase morbidity or mortality in emergency surgery when properly implemented. However, although our data is encouraging, the study might be underpowered to detect smaller differences which may affect safety if generalized to all patients. For example, the difference in superficial wound infection (12% vs. 16%) was not significant and a larger sample size might be needed to determine if there is truly no difference.

The successful implementation of ERAS in elderly PPU population challenges traditional beliefs about the feasibility of fast-track protocols in emergency surgery. Our results support the growing body of evidence, including work by Lohsiriwat [24], suggesting that age alone should not be a barrier to ERAS implementation when proper patient selection and protocol adherence are maintained. Elderly patients are particularly vulnerable to postoperative complications and prolonged recovery. Our study suggests that ERAS protocols are not only safe but also beneficial in enhancing recovery metrics in elderly patients undergoing emergency PPU repair.

The WSES guidelines for PPU emphasize the urgency of surgical repair and comprehensive perioperative care, particularly in elderly patients, to reduce morbidity and mortality [25]. Our ERAS-based approach aligns with these recommendations. Additionally, our structured perioperative strategy corresponds with the 2023 ERAS^®^ Society consensus guidelines for emergency laparotomy, reinforcing the safety and feasibility of enhanced recovery protocols even in urgent surgical settings [17].

This study has several strengths. First, to our knowledge, it represents the first comprehensive analysis of ERAS protocol implementation specifically in elderly patients undergoing emergency surgery for PPU. Our study included a comprehensive assessment of multiple outcome parameters, ranging from traditional recovery markers to detailed pain scores and functional recovery metrics. The homogeneity of the surgical technique is another point of strength. Additionally, the inclusion of a detailed ERAS protocol with specific elderly-focused modifications adds to the practical applicability of our findings in this high-risk group yielding multiple benefits without compromising patient safety. Furthermore, superior pain control and reduced opioid requirements are particularly relevant for elderly patients, who are more prone to opioid-related complications.

This study has several limitations. First, it was conducted at a single center; certain interventions (e.g., use of sevoflurane, TAP block, sugammadex for reversal) reflect institutional expertise and may not be feasible in all emergency settings, thus limiting the generalizability of the protocol. While the study focused on early recovery parameters, it did not assess long-term outcomes such as quality of life, functional independence after discharge, or complications beyond 30 days.

The implementation of ERAS as a new institutional initiative may have introduced performance bias (Hawthorne effect), whereby improved outcomes partly stem from increased attention, team motivation, or structured monitoring rather than the protocol itself. Additionally, our analysis excluded patients in refractory septic shock or those requiring open surgical conversion, meaning our findings apply only to a subset of hemodynamically stable patients eligible for laparoscopic and protocolized management. Therefore, caution is warranted in generalizing these results to more complex or unstable PPU cases.

Additionally, we acknowledge that the retrospective time-sequential design introduces the possibility of unmeasured confounding. Although the operations were performed by the same senior surgical and anesthesia team, but, improvements in our surgical technique, anesthesia practices, and perioperative care protocols over the 4-year period may have independently contributed to some of the observed benefits as mainly the latter cohort (ERAS group) benefited from this improvement.

Furthermore, part of the study period overlapped with the COVID-19 pandemic, which may have influenced hospital policies, discharge criteria, and resource allocation. Therefore, although the ERAS protocol was associated with improved outcomes, these results should be interpreted with caution as other secular factors could have played a role.

Due to the retrospective nature of the study, outcome assessors were not blinded. This introduces the potential for performance or assessment bias, particularly for subjective outcomes such as pain intensity and timing of mobilization. This limitation should be considered when interpreting the results.

Furthermore, although many outcome differences were statistically significant, not all are necessarily clinically meaningful. For instance, the observed difference in time to first flatus (2.34 vs. 2.53 days) represents less than 5 h, which is unlikely to affect clinical decision-making. In contrast, larger improvements in early mobilization and hospital stay are more impactful. Moreover, the study may have been underpowered to detect differences in rare outcomes such as anastomotic leakage or mortality. Additionally, functional recovery was assessed using basic clinical milestones (e.g., ambulation, oral intake), and validated tools such as the Barthel Index or ADL scores were not employed, which may limit comparability with studies using standardized recovery scales.

Future prospective randomized trials with longer follow-up periods and focusing on elderly-specific ERAS components will be of utmost significance.

## Conclusion

The implementation of ERAS protocols in elderly patients undergoing emergency surgery for PPU represents a significant shift in perioperative care. Our study provides evidence that ERAS protocols can be safely and effectively implemented in this high-risk population, yielding multiple benefits, in our cohort, including accelerated functional recovery, improved pain control, reduced opioid consumption, and shorter hospital stays, all without compromising patient safety or increasing complication rates. The success of ERAS implementation in this challenging scenario - emergency surgery in elderly patients - suggests that age and surgical urgency should not be considered absolute barriers to enhanced recovery protocols.

## Data Availability

The datasets used and/or analyzed during the current study are available from the corresponding author on reasonable request.
